# Reports on the Hospitals of the United Kingdom

**Published:** 1910-03-05

**Authors:** Henry Burdett


### REPORTS
ON THE
### Hospitals of the United Kingdom.

By SIR HENRY BURDETT, K.C.B., K.C.V.O.

### XXIX.—BLACKBURN AND EAST LANCASHIRE INFIRMARY.

This hospital is situated upon an elevated site which commands an abundance of sun and air. It was established in 1858, and has been enlarged three times since, and reconstructed, so far as the largest ward unit is concerned, quite recently. The floors of these wards have been laid with American willow and stained. We consider the staining a mistake, for the smartness and general appearance of the wards would be increased if the original wood was left plain and then polished.

The hospital contains 116 beds, with an average occupancy of 106. The wards are numerous, having four to twelve beds each, placed on either side of a wide central corridor. They are bright and comfortable, but their inconvenience will be realised when we state that there are no lavatories or baths to most of the smaller ones. This is a grave defect in the plan, and the hospital, like the Nurses' Home, seems to have suffered all along from the want of a directing mind to plan the whole institution and make each unit as perfect and easy to work as possible. There is a new out-patient department which contains an excellent casualty room with a small theatre. But the recovery rooms are too small for the amount of work which may have to be handled upon busy days. Everything appears to have been done to make the best of the existing buildings, except that new slop sinks and appliances and some up-to-date furniture are desirable in some of the wards. There is one grave structural defect, for th: slop sinks and lavatories of one of the wards hav: been placed in the operation theatre corridor. This blot should be removed without a moment's avoidable delay.

### Internal Management.

The general management of this hospital is good. The whole of the wards are spotlessly kept, and a careful inspection proved that there is the soul of order in the ward work. This is the more noteworthy as the furniture is none too modern or sufficient; in fact, several things might be supplied or done which Miss Gibbon could no doubt show would be helpful in the work and promote the general effectiveness of the wards. One good table in each ward, and an ample dressing trolley where most needed, might properly be supplied. In the County Hospital, Gloucester, some Governors have given a splendid oak ward table, which brightens the ward and is useful besides. Jones's table, if Jones would put his mind into it and see that it was good, might go down to posterity. Then Smith and Robinson might be moved to show what they could do, and so each ward should soon have all it requires.

The sisters and nurses do their work admirably, and King's and Guy's may be proud to know that the matron, Miss Gibbon, and the assistant matron, Miss Garside (to whom great credit is due for the present high state of efficiency and comfort apparent in the wards), were trained at King's and Guy's Hospitals respectively.

### The Nurses' Home.

As we have said, in view of the excellent site, if sufficient knowledge and thought had been put into this Home it should have been a model building; as it is, it exhibits an absence of the requirements of such a building. No grasp of the essential details necessary to utilise the available space to the best advantage, nor any well-thought-out plan which puts the rooms and their adjuncts upon an orderly basis so that their management may be simple and economical, is exhibited. It is not easy to reconstruct such a building as the present Nurses' Home, but the task should be taken in hand at once, for the excellence of the work of the sisters and nurses justifies greater comforts for the staff than are provided at present. It is no use having two rooms for recreation, both equally uncomfortable and neither suitably furnished. There should be one good recreation room, with an abundance of armchairs and couches, a piano, and a polished floor with rugs. It would be economical and better to have a kitchen for the Home where all the nurses' food is cooked, so that the cost of the nursing staff can be reduced to a minimum and the comfort and health of the nurses improved. The cost of this much-needed reconstruction should not be very large, and the funds justify the work being put in hand at once.

### Finance.

This hospital is in an unusually strong position financially, for the invested funds exceed £86,000. A voluntary hospital that has a reserve fund equal to five years' expenditure is safe against ordinary risks due to bad times. The ordinary expenditure is not quite £8,000 a year, so that £40,000 would amply cover such risks. It will be seen that the invested funds amount to more than double this sum. We point this out to show that the Managers need not delay the supply of various desirable things the wards now lack, or the necessary expenditure to bring the Nurses' Home up to date and provide all the additional accommodation in a reconstructed and enlarged Nurses' Home. These matters should engage the immediate attention of the committee, for they do not brook delay if justice to the staff and to the reputation of the infirmary are to be the first considerations. We are sorry to note the falling off in the receipts from the private nursing staff. We hope that steps may be taken to increase the private nursing staff, if the voluntary system is to prevail, as it ought to prevail for reasons too plain to need restatement here. Those voluntary hospitals which are wise maintain a private staff of adequate size to undertake the nursing of all sick cases which require the services of a private nurse. Then the committee could agree to give the first call on the private staff to the subscribers to the hospital. In this way not only should the existing subscription list be maintained, but the income from voluntary sources should be materially and promptly increased. By the institution of these important but relatively simple matters the infirmary should find itself in funds and with an ever-increasing revenue.

The **Kettering General Hospital** and the **Victoria Hospital, Frome,** will be reported on next week.

